# Temporal analysis of IgG antibody responses to *Plasmodium falciparum* antigens in relation to changing malaria epidemiology in a West African setting

**DOI:** 10.1186/s12936-017-1928-3

**Published:** 2017-07-11

**Authors:** Makhtar Niang, Oumy Niass, Nafissatou Diagne, Fatoumata Diene Sarr, Michel Matar Faye, Fode Diop, Babacar Diouf, Joseph Faye, Abdoulaye Badiane, Ronald Perraut, Cheikh Sokhna, Jean-François Trape, Adama Tall, Aissatou Toure-Balde

**Affiliations:** 1 Immunology Unit, Institut Pasteur Dakar, 36 Avenue Pasteur, BP 220, Dakar, Senegal; 2 Institut de Recherche pour le développement, BP 1386, Dakar, Senegal; 3 Epidemiology of Infectious Diseases Unit, Institut Pasteur Dakar, 36 Avenue Pasteur, BP 220, Dakar, Senegal

**Keywords:** Malaria, Transmission, Immunity, Controls

## Abstract

**Background:**

Coordinated scaled-up malaria control interventions have substantially contributed to the dramatic decrease of malaria-related morbidity and mortality in several endemic countries, including Senegal. However, the impacts of a given malaria control intervention on vector and parasite populations, acquired immunity, and disease burden remain very poorly documented largely due to the lack of continuous surveys. This study took advantage of the sera bank established as part of the Dielmo longitudinal project to investigate the dynamics of IgG antibody responses that accompanied the epidemiological changes resulting from malaria control interventions. Schizonts crude extract of a local strain of *Plasmodium falciparum* (Pfsch07/03) was used in ELISA to measure and compare seroprevalence and magnitude of IgG antibody responses from 2000 to 2012.

**Results:**

The prevalence of Pfsch07/03 IgG antibody responses progressively decreased from 97.25% in 2000 to 57.3% in 2012. The prevalence of Pfsch07/03 antibodies categorized between three different age groups (<7, 7–15, and >15 years) revealed increased seroprevalence with age ranging from 47.19 to 62.67 and 89.45%, respectively in (<7, 7–15, and >15 years) old age groups. A marked drop in seroprevalence was observed after 2008 and was significant in the younger (<7 years) and intermediate (7–15 years) age groups, unlike older individuals aged >15 years (p = 1.00).

**Conclusions:**

The study revealed a substantial contribution of all malaria control interventions to the decrease of IgG antibodies responses to Pfsch07/03 throughout prevention of human-mosquitos contacts, or reduction of parasite biomass. The present study demonstrates the wider potential of sero-epidemiological analysis in monitoring changes in malaria transmission resulting from a given malaria control intervention.

## Background

Owing to several combined strategies deployed by national malaria control programmes in malaria endemic countries, significant decrease in mortality and morbidity attributed to *Plasmodium falciparum* malaria has been recorded during the last decade [1]. In Senegal, the strategies included changes in the recommended first-line anti-malarial treatments with successive use of four first-line drugs regimens for treatment of malaria attacks from 1990 to 2010: oral quinine (June, 1990 to December, 1994), chloroquine (January, 1995 to October, 2003), sulfadoxine-pyrimethamine plus amodiaquine (SP + Am) (November, 2003 to May, 2006) and artesunate plus amodiaquine since June, 2006. These policies were reinforced by a systematic use of rapid diagnostic tests (RDTs) in 2007 and a countrywide deployment of long-lasting insecticide-treated bet nets (LLINs) since August 2008. These policies have substantially contributed in the dramatic decrease of both malaria morbidity and mortality in several Senegalese regions [2, 3].

To guide strategies to eliminate malaria from endemic areas, a better understanding of the effect of malaria control interventions on vector and parasite populations, acquired immunity, and disease burden is needed. Moreover, the impacts of implemented strategies deserve to be monitored overtime to anticipate the consequences of changing malaria epidemiology [4]. In most malaria endemic countries in Africa, the changes in the burden of *P. falciparum* disease over recent years have remained very poorly defined probably due to a lack of continuous surveys.

In Dielmo, an area of intense and perennial malaria transmission situated in the centre of Senegal [5] a longitudinal prospective study of malaria infection and the determinants of the disease in a community is conducted since 1990 [6]. The above mentioned control strategies, implemented by Senegalese National Malaria Control Programme, have been deployed at the time in Dielmo. The monitoring and analysis of malaria epidemiology in Dielmo over two decades revealed dramatic decrease of all malaria indicators (entomological inoculation rate, parasite prevalence changes, morbidity and mortality) between 1990 and 2012 [7]. The choice of first line antimalarial treatment and the deployment of LLINs to the entire population are believed to be the most important factors governing the dramatic changes in parasite rates and malaria morbidity [7].

It has been shown that antimalarial antibodies play an important role in the efficacy of anti-malarial drugs in younger children more susceptible to the disease [8]. Immunoglobulin G (IgG) can transfer protection and has been shown to contribute to treatment efficacy.

In light with this, a recent evaluation of the relationship between changes in malaria transmission and antibody responses to crude *P. falciparum* extracts between 2000 and 2010 showed a dramatic decline in seropositivity in Dielmo and Ndiop [9], two Senegalese villages where longitudinal follow-up have been conducted since 1990 and 1993, respectively [6, 7]. In both villages, the magnitude of antibody responses in seropositive individuals was significantly higher in 2000 than in 2010 [9].

To gain further insights on the impact of malaria control interventions on anti-malarial antibodies responses, the study closely investigated the dynamics of IgG antibodies responses to crude *P. falciparum* antigens in relation to changing malaria epidemiology in Dielmo from 2000 to 2012.

## Methods

### Population and sera collection

The study was conducted using sera collected from cross-sectional sampling in 2000, 2002, 2008, 2010 and 2012 from inhabitants of Dielmo village. The sera were withdrawn from the collection of archived biological specimens established as part of the Dielmo cohort study described elsewhere [6, 10]. This cohort study has been established in order to investigate the determinants of malaria infection and particularly those of the immunity to malaria. The different activities of this project include annual blood sampling that are archived allowing to study the evolution of biological parameters in relation with the evolution of malaria epidemiology. A total of 1235 sera collected from different years of the annually collected samples were included in this study: 218 in 2000, 186 in 2002, 269 in 2008, 288 in 2010 and 274 in 2012. The studied cohorts were constituted at periods of different malaria transmission intensity with entomological inoculate rates (EIR) progressively decreasing from 482.6 infected bites/person/year in 2000 to 0.3 infected bites/person/year in 2012 (Table 1).

**Table 1:** Demographic characteristics of the study population

	2000 N (%) 218	2002 N (%) 186	2008 N (%) 269	2010 N (%) 288	2012 N (%) 274	p value
EIR	482.6	409.9	12.3	2.2	0.3	
Treatment	Chloroquine	Chloroquine	ACT	ACT	ACT	
Sex						
Male	117 (53.67)	90 (48.39)	128 (47.58)	136 (47.22)	125 (45.62)	0.09
Female	101 (46.33)	96 (51.61)	141 (52.42)	152 (52.78)	149 (54.38)	
Age						
Mean	27.48	29.51	26.72	25.3	26.77	<0.001
[min–max]	[5–89]	[3.4–80.0]	[4.8–78]	[3.4–75.1]	[5.2–90.9]	
95% CI	[24.9; 30.0]	[26.7; 32.3]	[24.5; 28.9]	[23.2; 27.4]	[24.6; 29.0]	
Age groups (years)						
<7	16 (7.34)	15 (8.06)	20 (7.44)	24 (8.33)	14 (5.11)	<0.001
(7–15)	65 (29.82)	35 (18.82)	75 (27.88)	93 (32.29)	91 (33.21)	
>15	137 (62.84)	136 (73.12)	174 (64.68)	171 (59.38)	169 (61.68)	

Blood samples were collected at the end of the rainy season and sera were separated from blood pellets and stored at −20 °C until use for immunological studies. Informed consent for the study was obtained from the individuals, and/or their parents or legal guardians. Sociodemographic characteristics for the donors of these sera are given in Table 1. This study has been examined and approved by the Senegalese National Ethical Committee for Health Research.

### Setting

When follow-up programme of the Dielmo villagers was initiated in 1990, Dielmo was a malaria holoendemic area due to the presence of a small stream where anopheline mosquitoes bred all year round [5, 6, 11], thus sustaining an intense and perennial malaria transmission. Since 1990, the community of Dielmo has been the focus of intense monitoring and implementation of malaria control interventions simultaneously following at the same time their distribution in the country, in compliance with the national guidelines decided by the Senegalese Ministry of Health according to World Health Organization (WHO) recommendations.

### Crude schizont extract preparation from *Plasmodium falciparum* 07/03 strain

The adaptation and in vitro maintenance of the *P. falciparum* strain 07/03 (Pf07/03) isolated from a malaria-infected patient in Dielmo have been detailed previously [9, 12, 13]. Crude Pf07/03 schizonts extract (Pfsch07/03) was prepared following the method published by Wahlgren et al. [14]. After centrifugation of a highly synchronized Pf07/03 culture at approximately 10% parasitaemia, one volume of the pellet fraction was lysed in three volumes of sterile distilled water and vortexed for homogenization. The extract was frozen in nitrogen liquid in working aliquots.

Non-infected red blood cells were processed as described above and used as control in the ELISA to discriminate IgG antibodies responses to Pfsch0703 antigens from anti-erythrocytes antibodies (autoantibodies).

### Immunological assay by ELISA

ELISA Maxisorp plates (Nunc, Roskilde, Denmark) were used to optimize the dilution by dose effect. The optimal dilution was 1/300 and this was used for coating. Plates were coated with 100 μl of water-soluble crude extracts of Pfsch07/03. Non-infected red blood cells lysate was diluted in PBS and distributed into some wells on each plate as a control. The coated plates were incubated overnight at 4 °C and washed. Then, 200 μl of blocking buffer (2% BSA in PBS with 0.05% Tween) was added to each well and the plates incubated for 1 h at 37 °C. Serum samples were diluted at 1/200 in a dilution buffer (1% BSA in PBS with 0.05% Tween) and 100 μl of diluted serum was distributed into each well.

Negative and positive controls were included on each plate: positive controls were from Dielmo/Ndiop hyperimmune individuals and negative controls were from European individuals who have never been exposed to malaria. Polyclonal goat anti-human IgG conjugated to peroxidase at a dilution of 1/6000 in the dilution buffer (1% BSA in PBS-Tween 0.05%) was then added. Bound peroxidase was detected with orthotoluidine/H_2_O_2_ (100 μl) and the reaction was stopped by addition of 4 N H_2_SO_4_ (50 μl/well). Between each incubation phase, the ELISA plates were washed extensively with PBS-0.05% Tween. The optical density (OD) at 450 nm was read in a BIO-RAD Microplate Reader (iMark). The threshold for positivity was defined as an OD ratio >2 (OD sample/OD naive serum). Inter-assay variations for positive controls did not exceed 20%.

### Statistical analysis

Seroprevalence between age groups and years were compared using Pearson Chi-2 or Fisher Exact test. Non-parametrical test, Wilcoxon and Kruskall Wallis were used for analysis of level of antibody responses. The threshold of significance was fixed at 0.05. Thus a p value of 0.05 or less was considered as significant. The statistical analyses were performed using R software (version 3.0.2).

## Results

### Characteristics of the study population

A total of 1235 selected sera samples were distributed as follows: 218 in 2000, 186 in 2002, 269 in 2008, 288 in 2010 and 274 in 2012. The samples were obtained from inhabitants of Dielmo aged 3.4 to 90.9 years old and were tested for antibody responses to crude schizont extract from a locally adapted *P. falciparum* parasite strain (Pf07/03). The characteristics of the study population are summarized in Table 1. The mean age was 27.48 years [5–89], 29.51 years [3.4–80], 26.72 years [4.8–78], 25.3 years [3.4–75.1] and 26.77 years [5.2–90.9], respectively in 2000, 2002, 2008, 2010 and 2012. The sex ratio (M/F) was in favour of females except for 2000 (Table 1). For all five survey periods, the majority of individuals were aged 15 years and above (Table 1).

### Seroprevalence of Pfsch07/03 IgG antibodies across sampling period

**Fig. 1:**
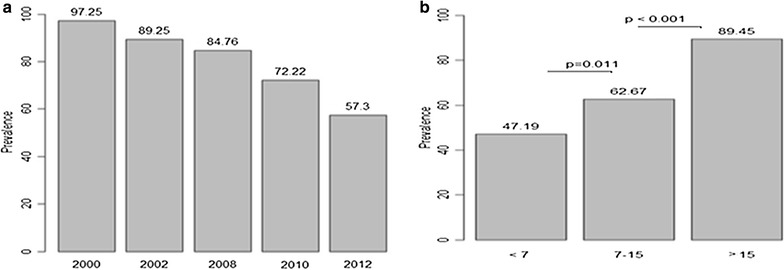
Prevalence of antibody responses to PfSch0703 categorized by sampling period (**a**) and age group (**b**)

The prevalence of Pfsch07/03 antibodies progressively decreased from 97.25% in 2000 to 57.3% in 2012 (Fig. 1a). The prevalence of Pfsch07/03 antibodies categorized between three different age groups (<7, 7–15, and >15 years) revealed increased seroprevalence with age ranging from 47.19 to 62.67 and 89.45%, respectively in (<7, 7–15, and >15 years) old age groups (Fig. 1b). The differences in the seroprevalence of Pfsch07/03 antibodies were significant between all age groups (Fisher’s Exact test, p < 0.05).

### Seroprevalence dynamics of Pfsch07/03 IgG antibodies according to age groups

**Fig. 2:**
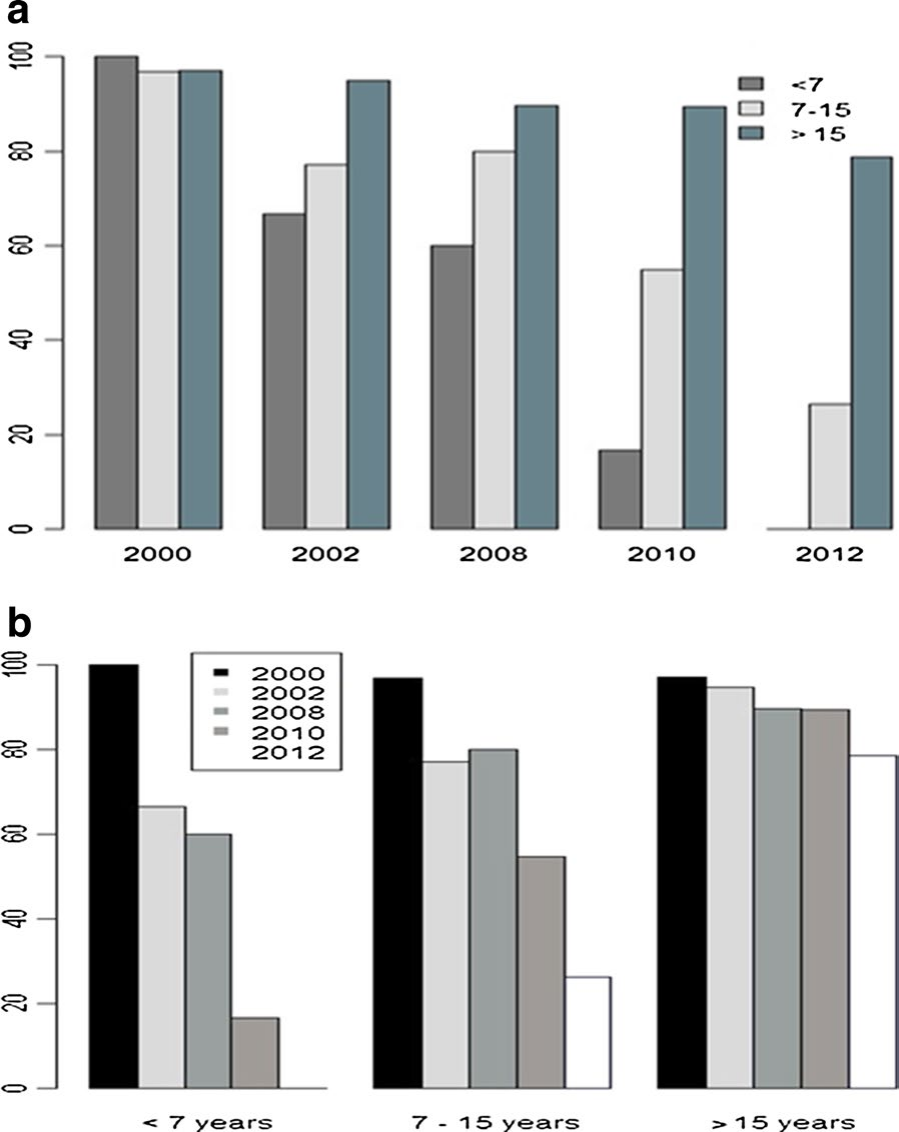
Dynamics of antibody responses to PfSch0703 analysed with collection period (**a**) and age groups (**b**)

The prevalences of Pfsch07/03 antibody responses were also categorized within the three defined age groups (<7, 7–15 and >15 years) for the five study periods. In 2000, the prevalence of Pfsch07/03 antibodies was high in all age groups (>95%) and was surprisingly highest in the youngest age group (100%) although the differences between age groups were not significant (p = 0.781) (Fig. 2a). For the following periods (2002–2012), seroprevalence of Pfsch07/03 antibodies showed a similar trend of increased positive responses with age groups (Fig. 2a). By contrast to 2000, the differences in seroprevalence between age groups for any given year were highly significant (p < 0.001).

The analysis of seroprevalence dynamics within age groups revealed three major tendencies. Firstly, in the younger age group (<7 years), seroprevalence progressively decreased from 2000 to 2010 and no reactivity was observed in 2012 (Fig. 2b). Comparative analysis of the seroprevalence dynamics between periods within this age group revealed statistically significant differences only between 2000 and 2002 (p = 0.042) and 2008 and 2010 (p = 0.0078) (Fig. 2b). Secondly, in the intermediate age group (7–15 years), besides a slightly non-significant increase of antibody responses from 2002 to 2008 (p = 0.92), the overall profile was similar to that observed with the younger age group though reactivity was observed in 2012 in the intermediate group (Fig. 2b). Statistical comparisons revealed significant differences between 2000 and 2002 (p = 0.005), 2008 and 2010 (p = 0.001) and 2010 and 2012 (p = 0.0001) (Fig. 2b). Lastly, in the older age group (>15 years), a progressive non-significant decrease of seroprevalence to Pfsch07/03 was observed from 2000 to 2010, followed by a statistically significant decreased seroprevalence between 2010 and 2012 (p = 0.01) (Fig. 2b).

Taken together, there was a marked drop in seroprevalence after 2008 (year of introduction of LLINs) that was significant in the younger (<7 years) and intermediate (7–15 years) age groups but not in the older one (>15 years) (p = 1.00).

### Dynamics of IgG antibodies levels according to malaria control interventions

**Fig. 3:**
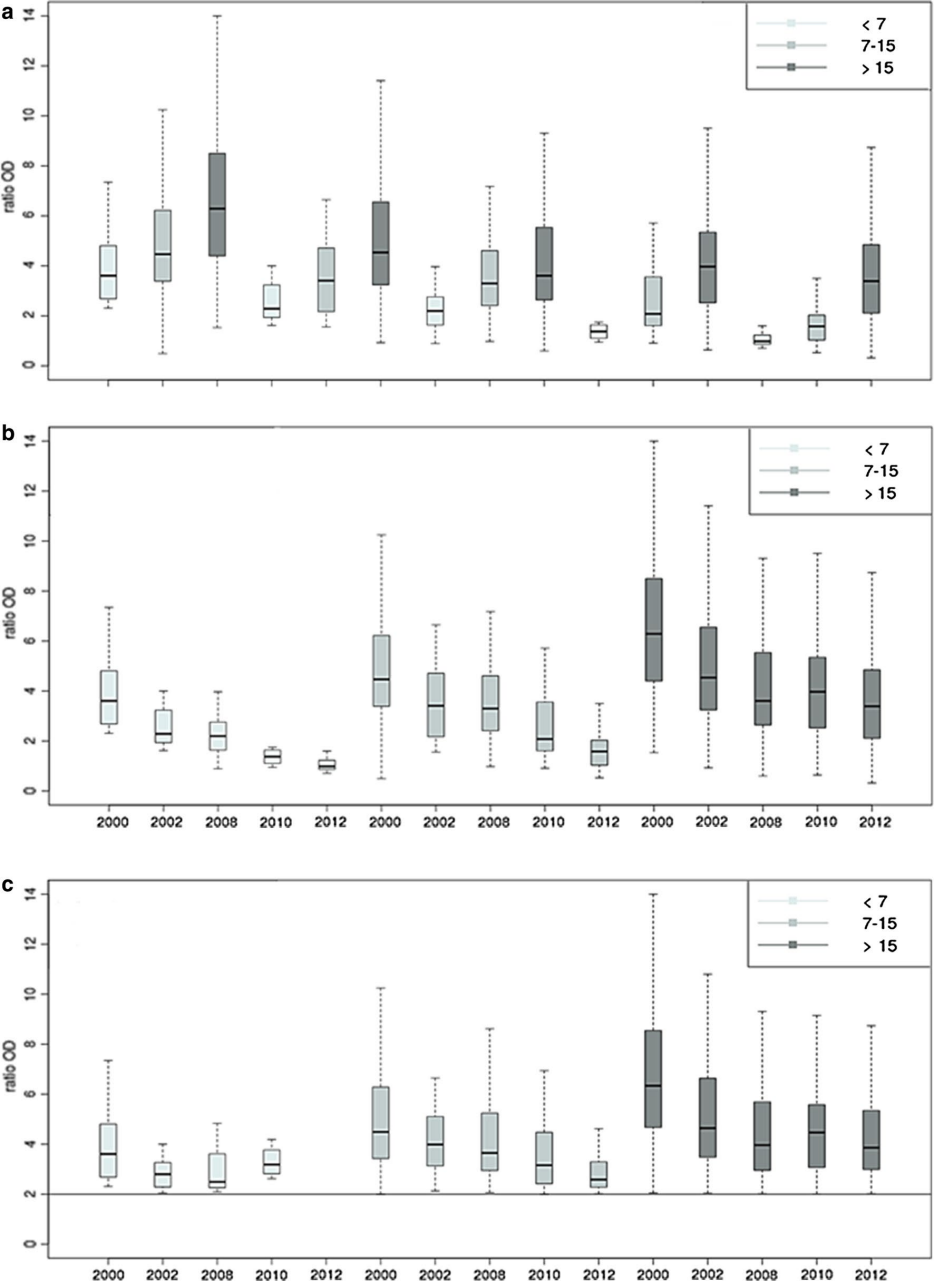
Level and dynamics of antibody responses to PfSch0703 by period and age group for all participants (**a**, **b**) and among positive responders (**c**)

The IgG antibody levels to Pfsch07/03 and their distribution were compared between categorized age groups for the different study periods. For any given period, Pfsch07/03 IgG antibody levels progressively increased with age (Fig. 3a). Several tendencies could be drawn when IgG antibodies responses from the different age groups were analysed in relation to malaria control interventions: From 2000 to 2002, a period of extensive use of chloroquine as first line antimalarial treatment, mean levels of Pfsch07/03 IgG antibody responses decreased for all age groups (Fig. 3a). A shift in the mean levels of IgG antibodies responses occurred between 2000 and 2002 with mean levels of IgG antibodies in 2000 for the <7 years and (7–15 years) age groups equaling those of the (7–15 years) and (>15 years) age groups respectively in 2002 (Fig. 3a). By contrast, no significant change in mean levels of IgG antibodies were observed between 2002 and 2008 in children (<7 years) (p = 0.050) and younger adults (7–15 years) (p = 0.953) whereas IgG antibodies levels significantly (p = 0.002) dropped in older individuals (>15 years).

From 2008 to 2010, the mean level of IgG antibody responses significantly dropped in the (<7 years) (p = 0.006) and (7–15 years) (p = 0.0001) age groups contrasting with the (>15 years) age group for which a non-significant increase (p = 0.92) of mean IgG antibody level was recorded (Fig. 3a). The mean level of IgG antibody decreased significantly in older individuals (>7 and 7–15 years) (p < 0.05) between 2010 and 2012 while no response was observed in the younger individuals in 2012.

The decrease IgG antibodies responses was significant (p < 0.05) in the (<7 and 7–15 years) age groups, for all year-to-year comparisons except between 2002 and 2008. The profile observed in older individuals (>15 years group) was broadly similar to that of the intermediate group with a unique non-significant decline recorded between 2008 and 2010 (p = 0.921). The data suggest that interventions such as the shift from CQ to SP + Am in 2003 and the deployment of ACT in 2005 have somewhat impacted on the magnitude of IgG responses to *P. falciparum* antigens and this was particularly apparent in the older age group (Fig. 3b).

While the magnitude of Pfsch07/03 IgG antibody responses in positive responders (OD ≥ 2) progressively decreased from 2000 to 2012 for the intermediate age group (7–15 years), the profiles were different for the younger and older age groups (Fig. 3c). In fact, in the younger age group (<7 years), the mean levels of positive IgG antibodies against Pfsch0703 decreased from 2000 to 2008 but increased in 2010 whereas no positive response was observed in 2012 (Fig. 3b). In the (>15 years) age group, besides the highly significant drop of the mean levels of positive IgG antibodies responses recorded between 2000 and 2002 (p < 0.001), mean levels of IgG antibodies broadly fluctuated between 2002 and 2012, suggesting a moderate to no relationship between the magnitude of IgG antibodies responses and malaria interventions.

## Discussion

Since 1990, a longitudinal prospective study of malaria infection and the determinants of the disease is being conducted in a community living in Dielmo, an area of Senegal with intense and perennial malaria transmission [6, 7, 15]. The study involving daily monitoring of malaria and non-malaria-related fevers, recording of malaria attacks and clinical and parasitological data along with monthly mosquito captures have generated a unique dataset, which allows a better understanding of the effect of malaria control interventions on vector and parasite populations and acquired immunity. This study took advantage of the sera bank established as part of the Dielmo project to investigate the dynamics of IgG antibody responses that accompanied the epidemiological changes resulting from malaria control interventions. Schizonts crude extracts of Pf07/03 strain were used in ELISA to measure and compare seroprevalence and magnitude of IgG antibody responses in inhabitants of Dielmo from 2000 to 2012.

The high seroprevalence to Pfsch07/03 antibody responses observed in 2000 is consistent with the high level of exposure in this region of perennial transmission [16]. The finding is supported by previous studies that reported a high seroprevalence and protective role of antibodies in the endemic Dielmo area [9, 11] and West African countries [17] prior the deployment of intensive malaria control interventions. While Diop et al. [9] reported a substantial decline in seroprevalence in Dielmo, presumably as a consequence of the anti-malarial strategies implemented since 2008 in Senegal [18], the findings reported in this study imply that all implemented malaria control interventions have somewhat impacted on the anti-malarial IgG antibody responses regardless of the specific contribution of a given intervention in the observed findings. Importantly, the decrease in seroprevalence observed throughout the study periods was more pronounced after 2008 (2010 and 2012) resulting to no positive responder in younger children (<7 years) in 2012. This correspond to the period following the massive deployment of LLINs in the entire population in 2008 associated with a substantial reduction of *Anopheles funestus* mosquitoes and a decreased of the entomological inoculation rates to its lowest value in 2012 when parasite carriage almost disappeared in Dielmo villagers [7]. The finding is also in accordance with the proposed scenario that in Dielmo, the very potent effects of LLINs, combined with effective clearance of parasites by ACT, has led to a major decrease in incidence of malaria attacks [19].

The low (47.19%) and high (89.45%) seroprevalences to Pfsch07/03 antibodies respectively in children and older adults could be the reflect of more previous exposures to malaria infection of older adults. Further support to this is the significant decrease in seroprevalence observed in younger individuals compared to older ones, probably reflecting a delayed acquisition of immune responses in younger individuals originated from disrupted exposure to malaria following introduction of LLINs in 2008. Moreover, there is a general agreement that the persistence of clinical immunity acquired during early childhood is dependent on sustained exposure, thus antibody responses decreases when exposure to malaria is discontinued [20, 21]. By contrast, the non-significant decline recorded in older individuals (>15 years) can be explained by a “memory effect” leading to the persistence of their immune responses as a consequence of a history of contact with the parasite as previously suggested by others [22, 23].

Similar to the prevalence of IgG antibody responses, the mean levels of Pfsch07/03 antibodies decreased substantially from 2000 to 2012 in almost all age groups despite two noticeable exceptions recorded between 2008 and 2010: a non-significant increase of mean IgG antibody levels in older individuals (>15 years) and an increase in mean antibody level in younger infants (<7 years). The decrease of mean IgG antibody level observed between 2000 and 2008 suggested that all implemented first line treatments contributed somehow to the reduction of available parasite biomass for mosquitos’ blood meal uptake that could have led to limited infectious contacts between infected mosquitoes and human hosts. In fact, it has been shown that EIR decreased from 482.6 to 155.3 infected mosquito bites/person/year from 2000 to 2008 in Dielmo, leading to a reduction of malaria prevalence from 70.6% in 2000 to 12.3% in 2008 in children and from 34.8% in 2000 to 9.0% in 2008 in adults [7]. In parallel, parasites rates have progressively decreased during the period of extensive use of chloroquine as first-line treatment (until October, 2003), probably in relation with the high incidence of malaria attacks and thus the high number of treatments received [7]. The shift from CQ to SP + Am treatment on November 2006 was followed by a decrease of parasites rates which was much more rapid and greater in young children than in older individuals. The decrease was much more dramatic following introduction of ACT treatment on June, 2006; and subsequently after implementation of LLINs in 2008 with parasite rates becoming very low in all age groups [7].

The observed shift in the mean levels of Pfsch07/03 IgG antibodies in the younger individuals in 2000 (corresponding to the intermediate (7–15 years) age group in 2002 and 2008 and older (≥15 years group in 2010) revealed an additional interesting feature of malaria transmission and seroprevalence: the percentage of positive responders became progressively low from 2000 to 2008 in all age groups but increased significantly in the younger children (<7 years in 2010) before becoming null afterward in 2012. While the progressive decrease of the percentage of positive responders could be suggestive of a loss of immune responses to Pfsch07/03 antigens that adults had had when they were younger as a result of limited human-parasite contact, the increase seropositivity observed in younger individuals in 2010 could be explained by the rebound in malaria morbidity as previously reported [19]. In fact, following the deployment of LLINs in Dielmo on August, 2008, resistance to pyrethroids augmented exposure of inhabitants to *Anopheles gambiae* s.l. infective bites, which in a community whose immunity had meanwhile declined [24, 25], led to a rebound in malaria morbidity because almost all infections became symptomatic in all age groups. The combination of LLINs with ACT was so effective at preventing malaria infections in all age groups that acquired immunity in older children and adults rapidly decreased, thus allowing new infections to be symptomatic and consequently rapidly detected and treated, but also favoring the re-establishment of human-vector contacts that contributed in boosting immune responses to *P. falciparum* malaria antigens.

## Conclusions

The study revealed that all malaria control interventions implemented in Dielmo substantially contributed to the decrease of Pfsch07/03 IgG antibody responses either by preventing human-mosquitos contact, or by reducing parasite biomass. The present study demonstrates the wider potential of sero-epidemiological analysis in monitoring changes in malaria transmission resulting from a given malaria control intervention.
